# Malignant Melanoma Incidence and Association with Arsenic

**Published:** 1983-10

**Authors:** R. Philipp, A. O. Hughes, M. C. Robertson, T. F. Mitchell

**Affiliations:** Lecturer in Community Medicine, University of Bristol; Senior Lecturer in Medical Statistics, University of Bristol; Research Associate, University of Bristol; Medical Student, University of Bristol

## Abstract

The aetiology of malignant melanoma is not wholly explained by exposure to sunshine. The present of arsenic in the soil has been suggested as a possible causative factor. Geographical clustering of malignant melanoma cases in the South Western Region is demonstrated for males and this clustering is associated with the distribution of arsenic in the soil. No evidence of geographical clustering is however found for females. This suggests possible sex-specific differences in the aetiology of malignant melanoma and indicates the need for further study of the association with arsenic.


### Malignant Melanoma Incidence and Association with Arsenic

**R. Philipp, M.F.C.M.I., A.F.O.M.**
Lecturer in Community Medicine, University of Bristol
**A. O. Hughes, MSc, MPhil.**
Senior Lecturer in Medical Statistics, University of Bristol
**M. C. Robertson, B.Sc., Hons.**
Research Associate, University of Bristol
**T. F. Mitchell**
Medical Student, University of Bristol

### SUMMARY

The aetiology of malignant melanoma is not wholly explained by exposure to sunshine. The present of arsenic in the soil has been suggested as a possible causative factor. Geographical clustering of malignant melanoma cases in the South Western Region is demonstrated for males and this clustering is associated with the distribution of arsenic in the soil. No evidence of geographical clustering is however found for females. This suggests possible sex-specific differences in the aetiology of malignant melanoma and indicates the need for further study of the association with arsenic.

### INTRODUCTION

Although sunlight is considered the principal causal factor in the aetiology of malignant melanoma there are inconsistencies in the evidence. For example, in Western Australia, incidence rates are highest in Perth and the south west corner of the state, rather than in the north where exposure of the population to ultra violet light might be expected to be highest. The rates are highest in residents of high social class areas, and in indoor rather than outdoor workers, (Holman, Mulroney and Armstrong, 1980). In the United Kingdom, although Wessex and the South Western Region have identical hours of sunshine, incidence rates for malignant melanoma are considerably higher in the South Western Region for both sexes (Swerdlow, 1979). Within the South Western Region the annual male and female malignant melanoma incidence rates for the years 1955–69, have a low correlation ($r=-0.07$) (Swerdlow, 1979).

Arsenic, by combining with sulphydryl groups in body tissues, interferes with pyruvate-oxidase activity and is associated with cancers of the skin (Sommers and McManus, 1953). It was suggested recently that because of this association, the high incidence rates for malignant melanoma in South West England may be explained by the distribution of arsenic in the environment (Clough, 1980; Evans, 1980). This speculation provides the basis for the analysis that follows where evidence has been sought for spatial clustering of malignant melanoma cases in relation to the geochemical distribution of arsenic in the South Western Region.

### METHOD

The age, sex, place of residence and year of registration were obtained for all cases of malignant melanoma registered with the South Western Regional Health Authority Cancer Registry between 1967 and 1976. Local Authority districts were selected for examination as these are the smallest geographical unit for which population data are readily available. The population data were obtained from the Office of Population Censuses and Surveys (O.P.C.S.) 1971 Census. This Census was taken near the midpoint of the study period and was thought to be sufficiently accurate to allow calculation of standardised incidence ratios (SIRs) for malignant melanoma.

The South Western Regional Health Authority comprises the county districts of Gloucestershire, Avon, Somerset, Devon and Cornwall. Notification of cancers in the Region may, however, not be complete. For example, treatment of patients with malignant melanoma who are resident in Gloucestershire is often undertaken in adjacent regions and these cases are not always notified to the South Western Regional Health Authority Cancer Registry. In addition, in the 1974 National Health Service reorganisation, significant boundary changes affected this part of the Region. For these reasons Gloucestershire County District was excluded from the study. Cases resident in Bath Local Authority District were also excluded as since 1974 this district has comprised part of the Wessex Regional Health Authority and cases would be notified to the Wessex Cancer Registry. The Isles of Scilly District was also excluded as arsenic levels for this district were not known.

The distribution of arsenic in the South Western Region was taken from the Wolfson Geochemical Atlas (Applied Geochemistry Research Group, 1978, pp. 22–23). This Atlas lists arsenic levels obtained by taking samples from stream sediments throughout the United Kingdom. A single sample was taken, on average, from each 2.5 km grid square throughout the U.K. These data were plotted on maps to a 1 : 2,000,000 scale, and a smoothing technique used to average individual sample values. Six empirical concentration intervals of arsenic content are given in the Atlas >0, >7, >15, >30, >70, >150 parts per million (ppm). For this analysis, these six concentration intervals were amalgamated to form two broad levels of arsenic content each of which comprised three of these concentration intervals. Geographical areas with arsenic levels 30 ppm and above constituted a group that were considered as 'high' arsenic areas. Those areas with less than 30 ppm were considered together as a group of 'low' arsenic areas. The arsenic level of 30 ppm was considered by the Ministry of Agriculture, Fisheries and Food as a reasonable division point for these groupings. Local Authority districts were placed in either the 'high' or 'low' arsenic category after visual inspection of the geochemical map together with Ordnance Survey maps showing Local Authority district boundaries. In order to classify the Local Authority districts, visual inspection included weighting the arsenic levels according to the major centres of population. This inspection was undertaken independently by four observers who compared their findings. Unanimous agreement was reached for 20 of the total of 26 Local Authority districts that were studied. The remaining six Local Authority districts were placed in either the 'high' or 'low' arsenic category after discussion between the four observers. With this method 17 Local Authority districts were labelled 'high' arsenic districts and nine were labelled 'low' arsenic. The standardised incidence ratios using the indirect method, were compared between areas using standardised errors of the estimated SIRs.

### RESULTS

The SIRs calculated for Avon, Somerset, Devon and Cornwall County districts are set out in Table 1. This table shows that for the aggregated years 1967 to 1976 there were statistically significantly fewer cases of malignant melanoma in males resident in Avon County District than in other county districts. No statistically significant differences between county districts were found for incidence ratios in females.

As noted earlier, the Local Authority districts had been classified by arsenic content of stream sediments. All such districts in Cornwall were 'high' arsenic areas, but the counties of Avon, Somerset and Devon included 'high' arsenic districts as well as 'low' arsenic districts. A significantly raised SIR for malignant melanoma was found in males resident in the aggregated 'high' arsenic Local Authority districts (Table 2). The SIR for females resident in aggregated 'high' arsenic districts was not significantly raised.

Local Authority districts in England and Wales are grouped by the O.P.C.S. on the basis of their social and economic characteristics, into six family clusters (Weber and Craig, 1978). Local Authority districts in the South Western Region fall within Family 1 (all suburban and growth areas), Family 2 (all rural and resort areas), or Family 4 (all service centres – often

**Table 1**

**Malignant Melanoma Distribution by County Districts: 1967–76**

| *Local Authority districts* | | *Number of cases* | | *Standardised incidence ratios* | |
|---|---|---|---|---|---|
| *Group* | *No.* | *Males* | *Females* | *Males* | *Females* |
| Avon | 5 | 76 | 221 | 74.4* | 89.5 |
| Somerset | 5 | 51 | 124 | 100.4 | 102.9 |
| Devon | 10 | 136 | 311 | 110.7 | 105.0 |
| Cornwall | 6 | 65 | 132 | 124.7 | 106.2 |
| Aggregate | 26 | 328 | 788 | 100.0 | 100.0 |

* $P<0.01$.

old established provincial cities). Standardised incidence ratios were calculated for each of these three groupings (Table 3). The SIRs in Table 3 show a statistically significant deficit of cases of malignant melanoma in males resident in Local Authority districts belonging to Family Cluster I. No significant differences were demonstrated for females. As Local Authority districts in the study belonging to Family I are only represented within Avon County, these findings are similar to those presented in Table I. When however, these data for Family I are combined with data for Family 4 to form broad 'urban' groups of males and females, neither the SIRs for this 'urban' group nor the SIRs for the Family 2 (all rural and resort areas) are significantly different from the expected. (Table 4).

Nineteen of the 26 Local Authority districts in this study are included in O.P.C.s Family 2 (rural and resort areas). These nineteen Local Authority districts were separated into a group of eleven districts, labelled 'low' arsenic areas, and another group of eight districts labelled 'high' arsenic areas. The SIRs

**Table 2**

**Malignant Melanoma Distribution by Arsenic Soil Levels (Grouped Local Authority Districts): 1967–76**

| *Local Authority districts* | | *Number of cases* | | *Standardised incidence ratios* | |
|---|---|---|---|---|---|
| *Group* | *No.* | *Males* | *Females* | *Males* | *Females* |
| 'Low' arsenic | 17 | 201 | 541 | 88.4 | 98.6 |
| 'High' arsenic | 9 | 127 | 247 | 126.1* | 103.2 |
| Aggregate | 26 | 328 | 788 | 100.0 | 100.0 |

* $P < 0.05$

**Table 3**

**Malignant Melanoma Distribution by Local Authority Districts Grouped as Family Clusters: 1967–76**

| *Local Authority districts* | | *Number of cases* | | *Standardised incidence ratios* | |
|---|---|---|---|---|---|
| *Group* | *No.* | *Males* | *Females* | *Males* | *Females* |
| Family 1 | 4 | 35 | 111 | 72.1* | 95.7 |
| Family 2 | 19 | 204 | 455 | 110.6 | 103.3 |
| Family 4 | 3 | 89 | 222 | 93.7 | 96.0 |
| Aggregate | 26 | 328 | 788 | 100.0 | 100.0 |

* $P < 0.05$

**Table 4**

**Malignant Melanoma Distribution by Grouped Urban/Rural Local Authority Districts: 1967–76**

| *Local Authority districts* | | *Number of cases* | | *Standardised incidence ratios* | |
|---|---|---|---|---|---|
| *Group* | *No.* | *Males* | *Females* | *Males* | *Females* |
| Urban | 7 | 124 | 333 | 86.4 | 95.5 |
| Rural and resort | 19 | 204 | 455 | 110.6 | 103.3 |
| Aggregate | 26 | 328 | 788 | 100.0 | 100.0 |

**Table 5**

**Malignant Melanoma Distribution in Rural and Resort Local Authority Districts by 'High'/'Low' Arsenic Soil Levels 1967–76**

| *Local Authority districts* | | *Number of cases* | | *Standardised incidence ratios* | |
|---|---|---|---|---|---|
| *Group* | *No.* | *Males* | *Females* | *Males* | *Females* |
| 'Low' arsenic Rural and resort | 11 | 111 | 287 | 98.0 | 105.6 |
| 'High' arsenic Rural and resort | 8 | 93 | 168 | 130.7* | 99.6 |
| Aggregate | 19 | 204 | 455 | 110.4 | 103.3 |

* $P<0.05$.

calculated for these areas are significantly raised for males resident in 'high' arsenic rural and resort Local Authority districts (Table 5). Similar findings were not demonstrated for females.

### DISCUSSION

This study has demonstrated an association between male cases of malignant melanoma and arsenic levels in stream sediments. The association is consistent with the suggestion that arsenic may be a causative factor in malignant melanoma. No statistically significant differences were found for male malignant melanoma incidence between urban and rural areas. Male residents of rural and resort areas with 'low' arsenic levels were found to be at no greater risk of developing malignant melanoma than residents of urban areas, although a statistically significant risk was demonstrated for males resident in 'high' arsenic rural and resort areas. These findings could be explained by greater exposure to the soil that residents of rural and resort areas are likely to have, as compared with residents of urban areas. A similar association was not found for females. If arsenic is causally associated with malignant melanoma, there may be differences in exposure between the sexes, or differences in effect.

Although malignant melanoma incidence rates can be calculated for each Local Authority district, it is unfortunate that sunshine data have not been recorded for each district. Thus, although incidence rates vary within the South Western Region, a possible association with sunshine cannot be investigated.

Although stream sediment sampling gives a reliable measure of geochemical arsenic, it is not accurate for total exposure. In the general environment arsenic is also present in water and released into the air from mining and in the smelting and refining of metal ores. Arsenic is also found in occupational environments as a hardening agent in the manufacture of lead shot and glass, as a constituent of copper alloys and bronze, as an ingredient of some solders, in the manufacture of rat poison, insecticides, fungicides and herbicides, as a growth promoter in pigs, and as a constituent of sheep dips. In all these occupations males are more likely to be employed than females and agricultural exposure may involve more intimate contact with arsenic than in other industrial undertakings.

Incidence rates for females in the South Western Region are more than twice those for males (Swerdlow, 1979). Therefore, the failure to demonstrate any association between melanoma incidence in females and arsenic levels in stream sediments, whilst not supporting the arsenic hypothesis, could be explained by the specific exposure of females, throughout the Region, to a common and as yet unidentified source of arsenic. Males, while maybe not so generally exposed, show lower rates, except perhaps for those few who have a high exposure to arsenic in the soil through employment in agriculture. Unfortunately this suggestion cannot be further explored as cancer registry data for occupation are incomplete. An attempt was made to complete the occupational data by examination of hospital records. A pilot review of 200 case notes showed however that occupation had not been recorded for two-thirds of the patients.

The suggestion that factors related to employment could be important in the aetiology of malignant melanoma is supported by recent studies; an excess relative risk has been shown for chemists employed in a high energy physics research laboratory (Austin et al, 1981), an excess of observed deaths among graduates and fellows of the Royal Institute of Chemistry has been reported (Burrows, 1980), and the observed deaths among oil refinery workers is higher than expected (Rushton and Alderson,

1981). Further studies of occupational and environmental factors are indicated to clarify the role of arsenic.

### ACKNOWLEDGMENTS

We thank the South Western Regional Health Authority Medical Statistics Bureau for access to their Cancer Registry data.

### REFERENCES

Applied Geochemistry Research Group Imperial College of Science and Technology, London, 1978. The Wolfson Geochemical Atlas of England and Wales. Oxford, Clarendon Press, pp. 22–23.

AUSTIN, D. F., SNYDER, M. A., REYNOLDS, P. J., BIGGS, M. W. and STUBBS, H. A. (1981). Malignant melanoma among employees of Lawrence Livermore National Laboratory. *The Lancet*, **2**, 712–716.

BURROWS, G. E. (1980). Health care of people at work: screening of workers in research laboratories. *J. of the Soc. of Occupational Medicine*, **30**, 164–168.

CLOUGH, P. (1980). Incidence of malignant melanoma of the skin in England and Wales. *British Medical Journal*, **1**, 112.

EVANS, S. (1980). Incidence of malignant melanoma of the skin. *British Medical Journal*, **1**, 403.

HOLMAN, C. D. J., MULRONEY, C. D. and ARMSTRONG, B. K. (1980). Epidemiology of pre-invasive and invasive malignant melanoma in Western Australia. *Int. J. of Cancer*, **25**, 317–323.

RUSHTON, L. and ALDERSON, M. R. (1981). An epidemiological survey of eight oil refineries in Britain. *British Journal of Industrial Medicine*, **38**, 225–234.

SOMMERS, S. C. and McMANUS, R. G. (1953). Multiple arsenical cancers of skin and internal organs. *Cancer*, **6**, 347–359.

SWERDLOW, A. J. (1979). Incidence of malignant melanoma of the skin in England and Wales and its relationship to sunshine. *British Medical Journal*, **2**, 1324–1327.

WEBBER, R. and CRAIG, J. (1978). Which local authorities are alike. *Population Trends*, **5**, 13–19.

